# DNA Methylation Variation Trends during the Embryonic Development of Chicken

**DOI:** 10.1371/journal.pone.0159230

**Published:** 2016-07-20

**Authors:** Shizhao Li, Yufei Zhu, Lihui Zhi, Xiaoying Han, Jing Shen, Yanli Liu, Junhu Yao, Xiaojun Yang

**Affiliations:** 1 College of Animal Science and Technology, Northwest A&F University, Yangling, Shaanxi, People's Republic of China; 2 School of Mathematics and Computer Science, ShanXi Normal University, Linfen, Shanxi, People's Republic of China; Inc, UNITED STATES

## Abstract

The embryogenesis period is critical for epigenetic reprogramming and is thus of great significance in the research field of poultry epigenetics for elucidation of the trends in DNA methylation variations during the embryonic development of birds, particularly due to differences in embryogenesis between birds and mammals. Here, we first examined the variations in genomic DNA methylation during chicken embryogenesis through high-performance liquid chromatography using broilers as the model organism. We then identified the degree of DNA methylation of the promoters and gene bodies involved in two specific genes (*IGF2* and *TNF-α*) using the bisulfite sequencing polymerase chain reaction method. In addition, we measured the expression levels of *IGF2*, *TNF-α* and DNA methyltransferase (*DNMT*) 1, 3a and 3b. Our results showed that the genomic DNA methylation levels in the liver, heart and muscle increased during embryonic development and that the methylation level of the liver was significantly higher in mid-anaphase. In both the muscle and liver, the promoter methylation levels of *TNF-α* first increased and then decreased, whereas the gene body methylation levels remained lower at embryonic ages E8, 11 and 14 before increasing notably at E17. The promoter methylation level of *IGF2* decreased persistently, whereas the methylation levels in the gene body showed a continuous increase. No differences in the expression of *TNF-α* were found among E8, 11 and 14, whereas a significant increase was observed at E17. *IGF2* showed increasing expression level during the examined embryonic stages. In addition, the mRNA and protein levels of *DNMTs* increased with increasing embryonic ages. These results suggest that chicken shows increasing genomic DNA methylation patterns during the embryonic period. Furthermore, the genomic DNA methylation levels in tissues are closely related to the genes expression levels, and gene expression may be simultaneously regulated by promoter hypomethylation and gene body hypermethylation.

## Introduction

In mammals, the epigenome, the status of which is directly related to its stability and environmental sensitivity, undergoes two rounds of elimination and reconstruction during gametogenesis and embryogenesis, respectively [1]. In the conventional static model, epigenetic stability is proportional to the amount of DNA methylation and histone modification [2]. These two developmental periods − germ cell generation and early embryonic development, which involve dynamic changes in the epigenome − are therefore vulnerable to the environment. Consequently, these two periods have become the focus of epigenetics studies [3,4].

At present, most epigenetics research studies focus on mammals, such as mice [5], pigs [6], and humans [7]. During mammalian pregnancy, the fetus is bonded to the mother through the placenta; thus, it is relatively easy to exert the effect of an external stimulus (such as the environment or nutrition) on embryonic development from the perspective of the mother [5,6]. However, in poultry, embryogenesis occurs during separation from the mother, which has resulted in fewer epigenetics studies in birds.

Methylation modifications of cytosine residues in DNA, particulary within cytosine-guanine dinucleotides, constitute important epigenetic mechanisms [8]. DNA methylation performs numerous functions. In general, methylation within gene regulatory elements suppresses gene expression [9,10], whereas methylation of gene-deficient regions is vital for maintenance of chromosome structure and integrity [11–13]. DNA methylation can also repress the movement of mobile elements, thereby defending against parasitic sequences in DNA [14]. Among the many functions of DNA methylation, the necessary connection between promoter methylation and gene silencing has yielded the most convincing evidence [13]. Moreover, the available body of evidence indicates that gene body methylation is a feature of transcribed genes [15] and is positively correlated with gene expression [16]. However, little is known regarding the role of the status of gene body methylation in gene expression.

DNA methylation is catalyzed by DNA methyltransferases (DNMTs). Dnmt1 is regarded as the most important maintenance methyltransferase in vertebrates and is responsible for restoring the methylated status of newly synthesized daughter strands [13]. DNMT3a and DNMT3b are *de novo* methyltransferases that perform non-overlapping functions at different stages of embryonic development. These three DNMTs all play important roles during embryogenesis.

Genome-wide DNA methylation maps have been reported for many organisms, including humans, Arabidopsis, silkworm and rice, but the methylation patterns of birds remains rarely studied. In the field of poultry epigenetics research, the examination of the variations in DNA methylation during embryogenesis is therefore crucial.

The present study was therefore undertaken to explore the variations in the genomic DNA methylation levels during all stages of embryonic development in broilers. Simultaneously, to examine the potential underlying mechanisms regulating the varying patterns, we studied the methylation status of the promoters and gene bodies of two specific genes (*TNF-α* and *IGF2*), which serve different functions during the embryogenesis process of birds. We also studied the expression of these two genes and DNA methyltransferases. The information obtained in this study will therefore be useful for understanding the embryonic DNA methylation patterns of birds, and the findings might help provide a theoretical foundation for poultry epigenetics.

## Materials and Methods

### Ethics statement

This study was conducted in strict accordance with the Regulations for the Administration of Affairs Concerning Experimental Animals (Ministry of Science and Technology, China, revised 2004). The protocol was approved by the Institutional Animal Care and Use Committee (College of Animal Science and Technology, Northwest A&F University, China). Each chick embryo used was electrically stunned before necropsy for sampling, and all other efforts were made to minimize suffering.

### Animals and sampling

#### Incubation

A total of 300 fertilized broiler eggs (average weight 63.4 ± 0.17 g, ranging from 59.0 g to 68.2 g) laid by Cobb 500 breeder hens were obtained from DaCheng Investment Group of Xianyang, Shannxi, China and were uniformly assigned to 5 incubator trays. The microcomputer automatic incubator (9TV-2A, Beijing LanTianJiao Electronic Technology Co., Ltd, Beijing, China) used to hatch the broilers was calibrated before hatching. The inner temperature of the incubator was controlled at 38.0–38.2°C from d 1 to 10, 37.8–38.0°C from d 11 to 18 and 37.5–37.8°C from d 19 to 21. The humidity was maintained at 45% to 65%. A 270° overturn of the eggs was conducted for 3 min every 2 h until d 19. On d 3 and 10, all the eggs were candled, after which the infertile and dead eggs were removed. The incubation period was 21 d.

#### Sampling

Six well-developed eggs were selected from every embryonic age. The whole embryo was sampled from embryonic ages (E) 2 to 7, and the livers, hearts and muscles were sampled from E 11 to 20. The tissues were taken within 10 min, snap-frozen in liquid N_2_ and stored at -80°C until further analysis.

### High-performance liquid chromatography (HPLC)

#### DNA extraction and hydrolysis

Genomic DNA was extracted from the embryonic tissues of the broilers using a classical phenol/chloroform/isoamyl alcohol [25:24:1(v/v/v)] protocol. Residual RNA was treated with RNAses A and T_1_ (Thermo Fisher Scientific, USA) at final concentrations of 80 μg/mL and 1500 U/mL, respectively, at 37°C for 1 h. The DNA was dissolved in TE buffer (10 mM Tris-HCl, 1 mM EDTA, pH 8.0) and stored at -80°C until analysis.

An improved procedure was established and performed for DNA hydrolysis [17–19]. Briefly, a 150-μL enzymolysis system was prepared as follows: approximately 20 μg of tissue DNA, one-tenth volume of 0.1 M ammonium acetate (pH 7.5), 10 μL of DNAse 1 (Thermo Fisher Scientific, USA), 15 μL of 10× DNAse 1 buffer (MgCl_2_), 10 μL of FastAP Thermosensitive Alkaline Phosphatase (Thermo Fisher Scientific, USA), 15 μL of 10× Alkaline Phosphatase Buffer, 5 μL of Exonuclease 1 (Thermo Fisher Scientific, USA) and 15 μL of 10× Exonuclease 1 Buffer were mixed, and double-distilled water was added to 150 μL. The mixture was then incubated at 37°C for 4–5 h. The results of agarose gel electrophoresis showed that the genomic DNA was digested to deoxynucleosides (S1 Fig). The product was filtered through a 0.22-μm nylon membrane filter (Alltech, Deerfield, IL) and stored at -20°C until analysis.

#### HPLC analysis

HPLC analysis was conducted with a Hitachi D-2000 Elite Chromatography system equipped with an Ultimate Polar-RP column (C18, 4.6 mm × 250 mm, 5 μm) (Welch Materials, Inc., Shanghai). Prior to use, the instrument was checked to confirm that it met the sensitivity defined by the manufacturer. A 20-μL portion of the hydrolyzed DNA solution was injected into the analytical column and thermostatted at 25°C. The separation of deoxycytidine (dC, Sigma), 5-methyl-2'-deoxycytidine (5-mdC, TCI) and three other major DNA bases were obtained by isocratic elution (S2 and S3 Figs). The mobile phase was a 0.1% (volume fractions of phases) phosphoric acid solution. All the prepared solutions were filtered through 0.45-μm membranes, and the mobile phase was degassed before injection into the HPLC. The mobile phase flow rate was 1.3 mL/min, and the run time was 45 min. The detection wavelength was set at 273 nm.

The limits of detection of dC and 5-mdC were 0.015 and 0.097 mg/L, respectively. The limits of quantification of dC and 5-mdC were 0.045 and 0.323 mg/L, respectively. The recovery rates of dC and 5-mdC were also determined (S1 Table).

#### Calculation of methylation ratio

Standard solutions of dC and 5-mdC were used to generate calibration curves at the same gradient spread: 50, 20, 10, 5, 2, 1 and 0.5 mg/L (S4 Fig). Then, the peak areas of dC and 5-mdC obtained from the chromatogram were converted to their doses: C_dC_ and C_5-mdC_. The genomic DNA methylation ratio (MR) was calculated using the following formula:

$$MR\left(\%\right)=100\times {\text{C}}_{5-\text{mdC}}/\left({\text{C}}_{5-\text{mdC}}+{\text{C}}_{\text{dC}}\right)$$

### Bisulfite sequencing PCR (BSP)

#### DNA preparation and sodium bisulfite treatment

Genomic DNA was extracted from the embryonic tissues of the broilers using a classical phenol/chloroform/isoamyl alcohol (25:24:1 [v/v/v]) protocol. DNA was treated with sodium bisulfite using the EZ DNA Methylation Kit (Zymo Research, USA) according to the manufacturer's protocol, except that the conversion temperature was changed to 62°C. The modified DNA samples were diluted in 10–15 μL of distilled water and served as templates for immediate PCR amplification.

#### BSP analysis

**Fig 1:**
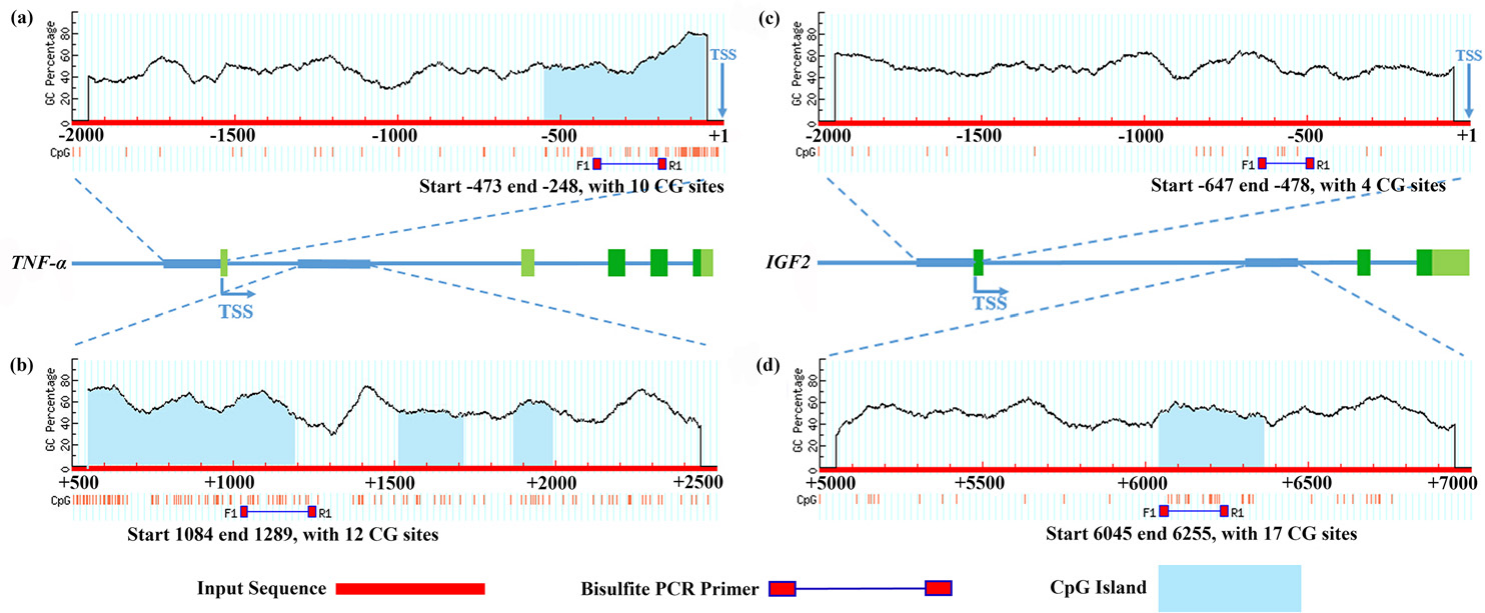
Schematic representation of the proximal promoter region and part of the first intron of tumer necrosis factor-alpha (*TNF-α*) and insulin-like growth factor 2 (*IGF2*) for predicting regions with a high GC content. The proximal promoter regions of *TNF-α* (a, -2000 to +1 base pairs [bp]) and *IGF2* (c, -2000 to +1 bp) and part of the first introns of *TNF-α* (b, +500 to +2500 bp) and *IGF2* (d, +5000 to +7000 bp) (modified output of MethPrimer program [20]) were used to predict regions of high GC content. A dashed black line indicates the GC percentage as represented on the y-axis, and the x-axis denotes the bp position on the *TNF-α* and *IGF2* 5'-untranslated and gene body regions. The arrows indicate the transcriptional start sites (TSS). The coordinates are given in relation to the translation initiation site (shown as +1); vertical red lines indicate the relative positions of CpG dinucleotides; solid lines depict the locations of the PCR primers. Input sequences are shown as the bold region of the intermediate solid blue line; the untranslated and translated exons of *TNF-α* and *IGF2* are indicated by light blue and dark blue bars, respectively. The start sites, end sites and numbers of CG loci of the PCR products are all shown under each bisulfite PCR primer.

A schematic representation of the detected sites for BSP in *TNF-α* and *IGF2* is shown in Fig 1. The BSP primers were designed using online MethPrimer software [20]. The sequences of the PCR primers used to amplify the targeted products are shown in Table 1. PCR was performed in a 20-μL reaction volume containing 100 ng of modified genomic DNA, 1 μL of each primer (10 μM/L), 2 μL of MgCl_2_ (25 mM), 0.8 μL of a 10 mM dNTP mixture, 2 μL of 10× PCR buffer, 0.2 μL of Taq DNA polymerase (TaKaRa, Dalian, China) and 12 μL of nuclease-free water. PCR was performed with a DNA Engine Thermal Cycler (Bio-Rad, USA) using the following program: 3 min at 95°C followed by 35 cycles of denaturation for 30 s at 95°C, annealing for 30 s at 62°C and extension for 30 s at 72°C and a final extension at 72°C for 5 min. Five separate amplifications were performed for each DNA sample, and the PCR products were gel-purified using a Gel Purification Kit (TaKaRa). The purified fragments were subcloned into the pMD19-T vector (TaKaRa) and used for the transformation of competent *Escherichia coli*. The cells were plated on LB containing 100 μg/mL ampicillin, 40 μL of 40 mg/mL X-gal (5-bromo-4-chloro-3-indolyl-β-D-galactopyranoside) and 4 μL of 20 mg/mL IPTG and were stored overnight in an incubator with a constant temperature of 37°C. The PCR product was inserted into the *lacZa* gene within the vector, allowing the use of X-gal to help distinguish the white colonies that were transformed with the plasmid with the ligated PCR product from the blue colonies that were transformed with empty plasmids. The experimental details described above were previously reported [21]. To analyze the transformants, 30 different positive clones for each subject were randomly selected and grown overnight in 2–5 mL of LB broth containing 100 μg/mL ampicillin on a shaking table at 37°C and 180 rpm. Then, 200–500 μL of LB broth was collected from each sample for sequencing (Sangon, Shanghai, China). The final sequencing results were processed with online QUMA software [22]. To control for sequence quality, we adopted a lower limit for identity of 90% and an upper limit of mismatches equal to 10 as default values and set 95% as the lower limit for the conversion efficiency and 5 as the upper limit of the number of unconverted cytosines as additional default values [22].

**Table 1:** Primer sequences for bisulfite sequencing polymerase chain reaction analysis.

Gene	Location	Accession number	Primer sequences	CpG loci	Annealing temperature (°C)	Fragment size (bp)
*TNF-α*	-473/-248	NC_006101.3	F:TAAGTTTTTTGGGGTTGAATTTAAT	10	62	226
			R:CAACTTCCCTACCAACTACAATAAC			
*TNF-α*	1084/1289	NC_006101.3	F:GAGAGTTGAAATTTTTTTGAGTTGA	12	58	206
			R:CAACAAAAAATATAAAAATAAAACC			
*IGF2*	-647/-478	NC_006092.3	F:TGGTTGTGTTGTAGATTTTTTTTGT	4	62	170
			R:ACACTAAATTTCACCTCCCATTTT			
*IGF2*	6045/6255	NC_006092.3	F:GTTGTTTTATTTGGTAAAATTTAGT	17	62	211
			R:TCCTATTACTCCTTAACAAACCCAA			

#### Real-time PCR for mRNA quantification

Total RNA was extracted from the tissue samples using a total RNA extraction kit (9767, Takara) according to the manufacturer’s protocol. The total RNA was quantified using a NanoDrop^®^ ND-1000 spectrophotometer (Thermo Scientific) with the OD value set at 260 nm, and the purity was assessed by determining the OD_260_/OD_280_ ratio using formaldehyde-agarose gel electrophoresis. cDNA was synthesized with a PrimeScript^®^ RT reagent Kit (Takara) according to the manufacturer’s protocols. All cDNA samples were stored at -20°C until use. The gene (*β-actin*, *TNF-α*, *IGF2*, *DNMT1*, *DNMT3a and DNMT3b*) expressions levels were determined using SYBR^®^ Premix Ex Taq^TM^ II (RR8R, Takara). Briefly, a 25-μL PCR mixture was prepared from 12.5 μL of SYBR^®^ Premix ExTaq II (2×), 1 μL of forward primer (10 μM/L), 1 μL of reverse primer (10 μM/L), 1 μL of cDNA, and 9.5 μL of double-distilled water. Primers for real-time PCR were synthesized by Sangon Biotech and are listed in Table 2. The PCR was amplifications were conducted in an iCycler iQ5 multicolor real-time PCR detection system (Bio-Rad Laboratories) programmed as follows: 95°C for 10 min; 40 cycles of 95°C for 10 s, 60°C for 30 s, 72°C for 30 s; and 72°C for 5 min. All samples were examined in triplicate, and the average cycle threshold (Ct) values were used for quantification via the 2^-ΔΔ*Ct*^ method [23].

**Table 2:** Primer sequences for quantitative real-time PCR analysis.

Gene	Accession number	Primer sequences	Fragment size (bp)
*β-actin*	X00182.1	F: ATTGTCCACCGCAAATGCTTC	114
		R: AAATAAAGCCATGCCAATCTCGTC	
*TNF-α*	AY765397.1	F: TGTGTATGTGCAGCAACCCGTAGT	229
		R: GGCATTGCAATTTGGACAGAAGT	
*IGF2*	NM_001030342.1	F: AGACCAGTGGGACGAAATAACA	121
		R: CACGCTCTGACTTGACGGAC	
*DNMT1*	NM_206952.1	F: ACAGCCTTCGCCGATTACA	248
		R: CTCTCCACCTGCTCCACCAC	
*DNMT3a*	NM_001024832.1	F: GGATAGCCAAGTTCAGCAAAG	145
		R: GGGAAGCCAAACACCCTCT	
*DNMT3b*	NM_001024828.1	F: GTGCTGTGCCTTGAACATTG	125
		R: TTCGTAACTTCGGAAACCATT	

DNMT: DNA methyltransferase

### Western blotting analysis

The total protein from 50 mg of frozen tissue samples was extracted using basic lysis buffer (50 mM Tris-HCl, 150 mM NaCl, 5 mM EDTA, 1% Triton X-100, and 0.1% SDS). The protein concentrations were measured using the Pierce^TM^ BCA Protein Assay Kit (Thermo Scientific, USA). A Western blotting analysis of the target proteins was performed according to the protocols provided by the manufacturer. Detailed information of the primary antibodies used in the Western blotting analysis in provided in S2 Table (online). β-actin was selected as the loading control for the total tissue protein.

### Statistical analysis

All the data were analyzed with one-way ANOVA using the GLM procedure with SAS software (version 8.02, SAS Institute Inc., Cary, NC). Significant differences between treatment groups were determined using Fisher’s least significant difference. The results are presented as the means with standard errors (means ± SE). Differences in treatment means were considered significant at *P* < 0.05, and significant levels of 0.05 < *P* < 0.10 were considered as trends.

## Results

### Genomic DNA methylation in the chicken embryo

**Fig 2:**
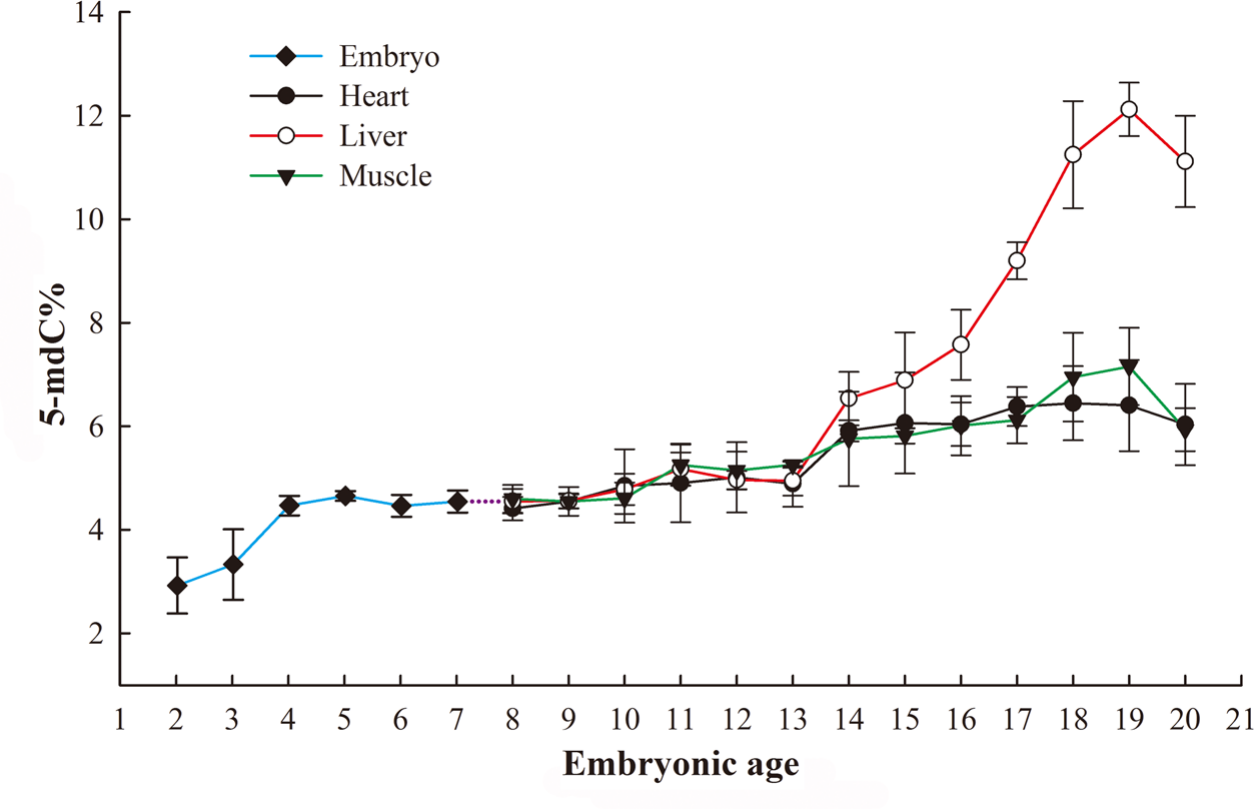
Variations in the genomic DNA methylation levels in broilers during the embryogenesis. The samples examined from embryonic ages (E) 2 to 7 were a mixture of the whole embryo; from E 9 to 20, the examined samples were separated into the heart, liver and muscle tissues. 5-mdC: 5-methyldeoxycytosine.

The genomic DNA methylation levels in the heart, muscle and liver increased with increasing embryonic age (Fig 2). From E2 to 4, the genomic DNA methylation levels of the embryo increased rapidly (3% to 5%); from E4 to 7, the methylation levels in the whole embryo remained steady; from E8 to 13, the methylation levels in the three tissues reached a stable level of approximately 5%; after E13, the genomic DNA methylation levels in the heart, muscle and liver showed a rapid increase, and the growth rate of these levels in the liver was markedly higher than those of the methylation levels in the heart and muscle; and the methylation levels in the tissues reached a peak at E19 (with a remarkable level of 12.1% in the liver) before decreasing at E20.

### Methylation of *TNF-α* and *IGF2* in the muscle and liver of the chicken embryo

**Fig 3:**
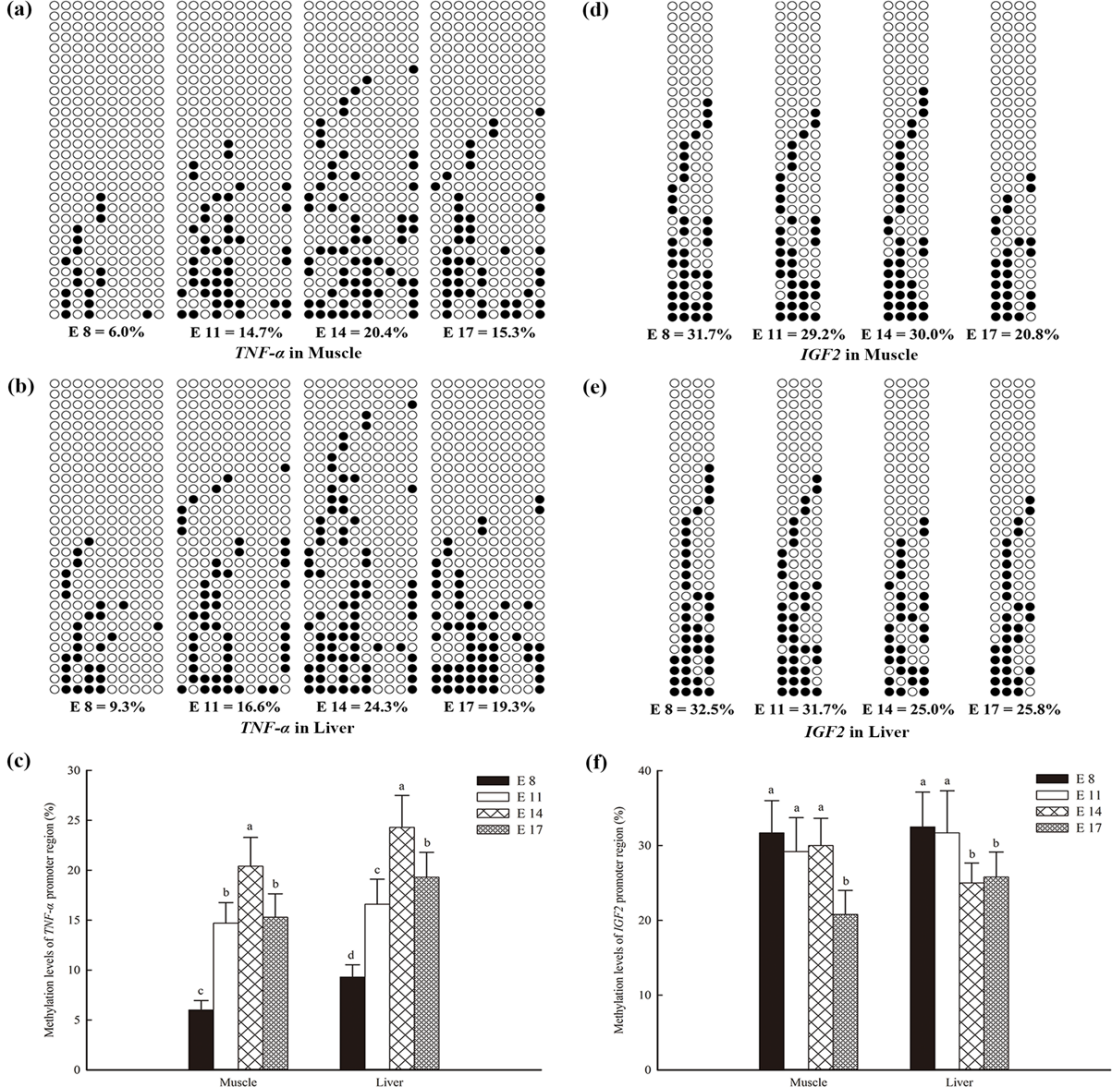
DNA methylation patterns of the tumer necrosis factor-alpha (*TNF-α*) and insulin-like growth factor 2 (*IGF2*) promoters in the muscles and livers of broilers. The analytic method was the bisulfite sequencing polymerase chain reaction. Each line represents an individual bacterial clone, and each circle represents a single CpG dinucleotide. Open circles indicate unmethylated CpGs, and black circles indicate methylated CpGs. (a) *TNF-α* detected in the muscle. (b) *TNF-α* detected in the liver. (d) *IGF2* detected in the muscle. (e) *IGF2* detected in the liver. (c) and (f) The methylation levels of the *TNF-α* and *IGF2* promoter regions in the histogram. The data are presented as the means with their standard errors (n = 6). Bars with different letters differed significantly (*P* < 0.05).

The methylation levels of the *TNF-α* promoter in the muscle initially increased (from E8 to E14) and then decreased (at E17) (*P* < 0.05; Fig 3(A) and 3(C)). Similarly, the promoter methylation level of *TNF-α* in the liver was higher at E14 (*P* < 0.05) than at E8, E11 and E17 (Fig 3(B) and 3(C)). For *IGF2*, the promoter methylation level in the muscle was lower at E17 (*P* < 0.05) than that at the other three embryonic ages; however, no differences were found among E8, E11 and E14 (Fig 3(D) and 3(F)). In the liver, the methylation levels of the *IGF2* promoter at E 14 and E 17 were significantly lower (*P* < 0.05) than those at E8 and E11; however, no differences were found between E8 and E11 or between E14 and E17 (Fig 3(E) and 3(F)).

**Fig 4:**
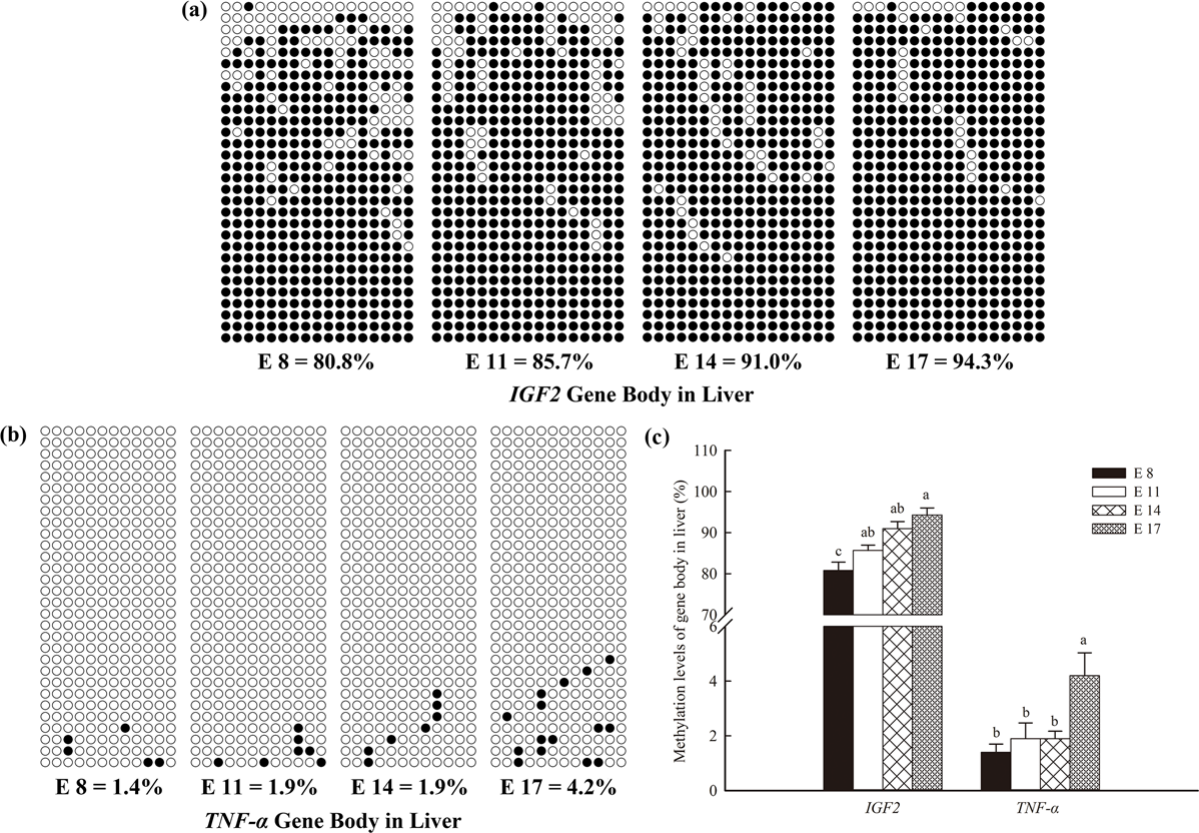
DNA methylation patterns of the tumer necrosis factor-alpha (*TNF-α*) and insulin-like growth factor 2 (*IGF2*) gene bodies in the livers of broilers. The analytic method was the bisulfite sequencing polymerase chain reaction. Each line represents an individual bacterial clone, and each circle represents a single CpG dinucleotide. Open circles indicate unmethylated CpGs, and black circles indicate methylated CpGs. (a) *TNF-α* detected in the liver. (b) *IGF2* detected in the liver. (c) The methylation levels of the *TNF-α* and *IGF2* gene bodies in the histogram.

**Fig 5:**
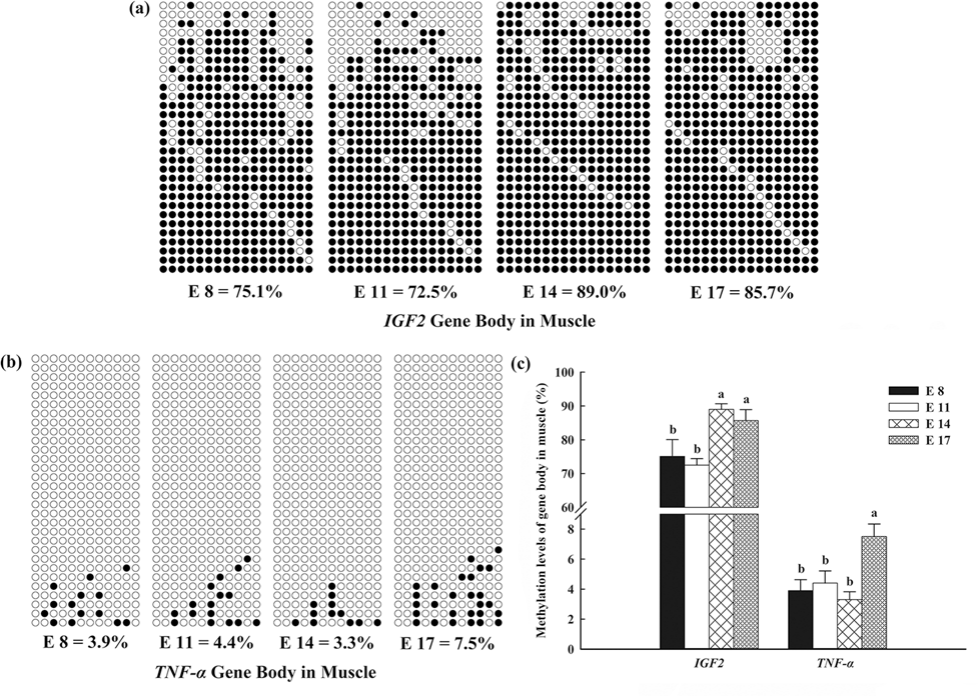
DNA methylation patterns of the tumer necrosis factor-alpha (*TNF-α*) and insulin-like growth factor 2 (*IGF2*) gene bodies in the muscles of broilers. The analytic method was the bisulfite sequencing polymerase chain reaction. Each line represents an individual bacterial clone, and each circle represents a single CpG dinucleotide. Open circles indicate unmethylated CpGs, and black circles indicate methylated CpGs. (a) *TNF-α* detected in the muscle. (b) *IGF2* detected in the muscle. (c) The methylation levels of the *TNF-α* and *IGF2* gene bodies in the histogram.

In both the livers and muscles, the methylation levels of the *TNF-α* gene body remained stably low at E8, E11 and E14, but notably increased (*P* < 0.05) at E17 (Figs 4(B), 4(C), 5(B) and 5(C)). In contrast, the methylation levels of the *IGF2* gene body in the livers increased continuously with increasing embryonic age (*P* < 0.05; Fig 4(A) and 4(C)), and the methylation levels at E14 and E17 in the muscle were significantly increased compared with those at E 8 and E 11.

### Muscular and hepatic expression of *TNF-α*, *IGF2* and *DNMTs*

**Fig 6:**
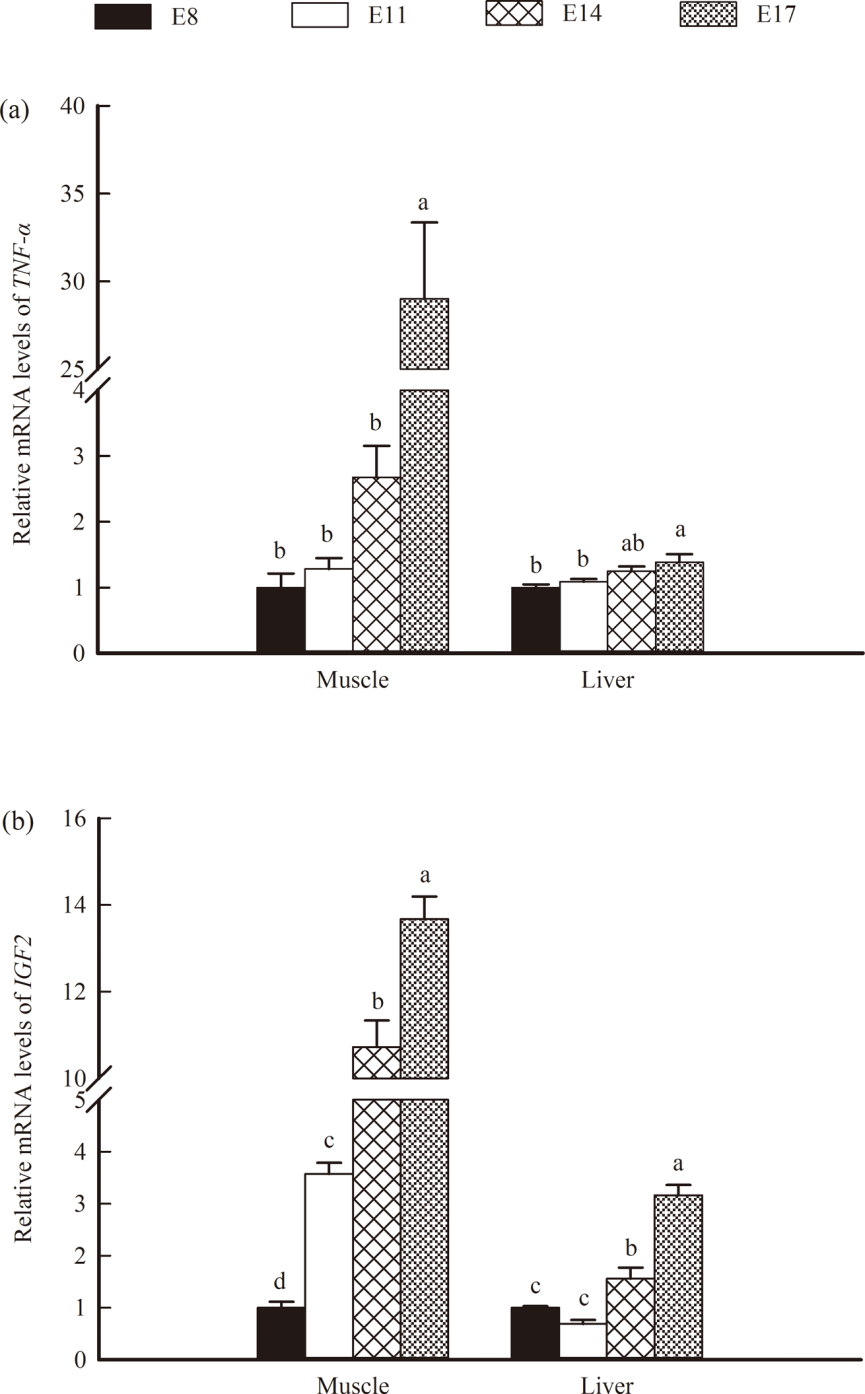
Relative mRNA levels of tumer necrosis factor-alpha (*TNF-α*) and insulin-like growth factor 2 (*IGF2*) in the muscles and livers of broilers. (a): *TNF-α*, (b): *IGF2*. The data represent the means with standard errors (n = 6). Bars with different letters differed significantly (*P* < 0.05).

**Fig 7:**
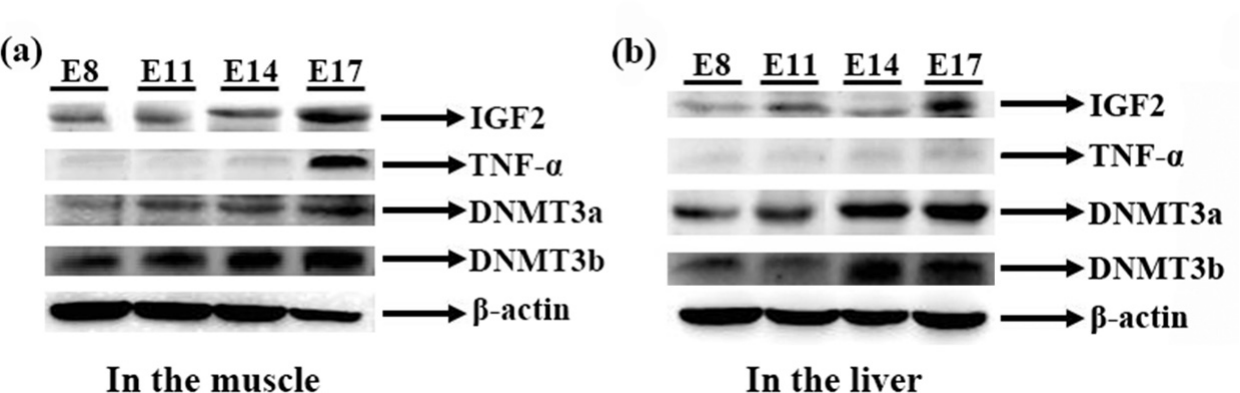
Protein expression levels of insulin-like growth factor 2 (IGF2), tumor necrosis factor-alpha (TNF-α) and DNA methyltransferases (DNMT) in the muscles and livers of broilers. (a) Protein expression levels of the target proteins in the livers. (b) Protein expression levels of the target proteins in the muscles.

No differences were found in the expression of *TNF-a* at E8, E11 and E14 in the muscle, but a predominant increase (*P* < 0.05) was detected at E17 (Figs 6(A) and 7(A)). In the liver, the mRNA and protein levels of *TNF-a* at E17 were higher than those at E8 and E11 but did not differ significantly from those at E14 (Figs 6(A) and 7(A)). The expression of *IGF2* showed a pronounced increase (*P* < 0.05) with increasing embryonic age, and the mRNA and protein levels of *IGF2* at E17 were higher (*P* < 0.05) than those at E 8, E 11 and E 14 (Figs 6(B) and 7(B)).

**Fig 8:**
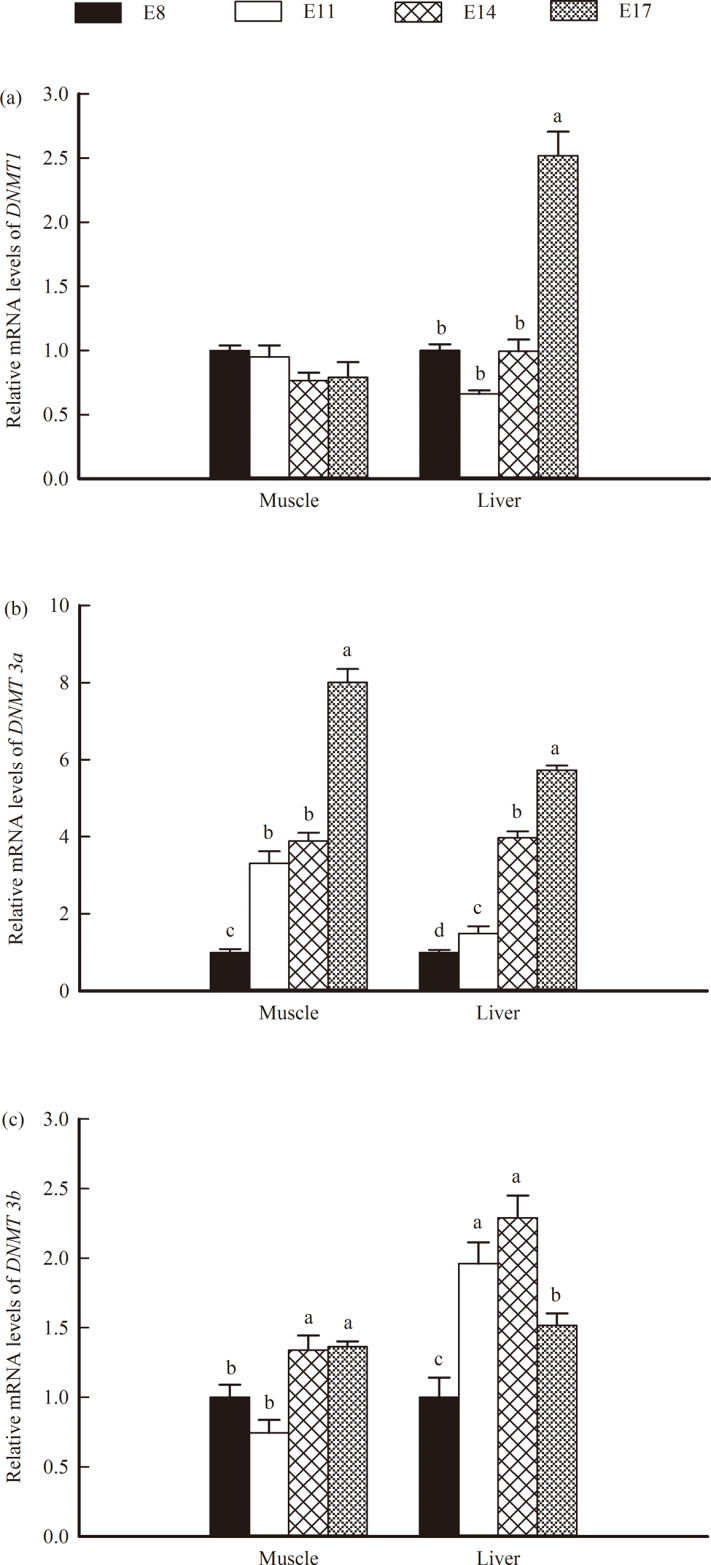
Relative mRNA levels of DNA methyltransferases (*DNMT*) 1, *DNMT3a* and *DNMT 3b* in the muscles and livers of broilers. (a): *DNMT1*, (b): *DNMT3a*, (c): *DNMT 3b*. The data represent the means with standard errors (n = 6). Bars with different letters differed significantly (*P* < 0.05).

In the liver of the chicken embryo, the highest mRNA level of *DNMT1* was detected at E 17, whereas no significant differences in the mRNA expression of this gene was found among E 8, E 11 and E 14 (Fig 8 (A)). The expression of *DNMT1* in the muscle of the chicken embryo showed no difference among the four embryonic ages. In both the muscle and the liver, the expression of *DNMT3a* exhibited an increase with increasing embryonic age (*P* < 0.05; Figs 7(B) and 8(B)). In addition, the expression of *DNMT3b* was increased at E14 and E17 (*P* < 0.05; Figs 7 (A) and 8(C)); in the liver, the *DNMT3b* mRNA and protein levels at E11 and E14 were higher than those at E 8 (*P* < 0.05), but a significant decrease was noted at E17 (*P* < 0.05; Figs 7 (B) and 8(C)).

## Discussion

To obtain an overview of the variations in DNA methylation during embryogenesis in broilers, we first examined the genomic DNA methylation status in various tissues. In general, our results indicated that the DNA methylation patterns of the chicken embryo showed a gradually increasing tendency. The embryonic development of chicken can be separated into three stages: from E1 to 4, the internal organs (such as the heart, liver, and gonads) experience rapid differentiation; from E 5 to 15, the external tissues (such as the skeleton and muscle, which have relatively singular functions) grow rapidly but to not become fully functional; and from E16 to 19, the embryonic mass increases rapidly, and the tissues and organs become fully functional for hatching [24–26]. Surprisingly, the different embryonic stages of the fetus agree with the different stages (E2 to 4, E4 to 13 (E4 to E7 for the whole embryo and E8 to E13 for the three tissues), and E13 to 19) detected in the variations in the genomic DNA methylation status observed in the present study. Another interesting finding of the present study is that the muscle, heart and liver exhibited decreases in genomic DNA methylation after E19. In commercial hatchery, eggs are moved to trays at E19, and the allantoic respiration of the chick begins to transition to pulmonary respiration at this stage [27,28]. Pipping chicks are then faced with new surroundings, including a different temperature, an altered humidity and the presence of viruses in the air. Theses external pressures may be associated with the decrease in genomic DNA methylation observed at E20, although further studies are necessary to reveal the underlying cause.

5-methyl-2'-deoxycytidine (5-mdC) is widely observed in vertebrate DNA [29], and the levels of 5-mdC in genomic DNA present changes during developmental process that follow tissue-specific patterns [30]. In this study, the DNA methylation status of the liver in the chicken embryo was distinctly different from that of the heart and muscle and reached a remarkably high level of 12.1% at E19. The DNA methylation patterns during the later phase of the embryonic development of chickens revealed that the jejunum showed a 5-mC level of 11% at E14 [31]. Genome-wide mapping of DNA methylation in chicken showed that the methylated CpGs in the liver tissue of the red jungle fowl and avian broiler were 5.7% and 9.1%, respectively [32]. Previous studies have also indicated that the genomic DNA methylation patterns of different tissues in the same animal exhibit differences [33,34], and the peak in the genomic DNA methylation level detected in the late stage of embryonic development in the present work is likely connected to the increased embryonic weight [35].

To explore whether the DNA methylation patterns of specific genes in various tissues were the same as the genomic DNA methylation patterns, we first conducted a deeper analysis of the methylation status of the promoters of two specific genes (*TNF-α* and *IGF2*), which exert different functions during bird embryogenesis. TNF-α plays an important role in anti-infection immunity [36–38]. The precondition for the functional display of TNF-α is stimulation by an external antigen [39]. Due to obstruction of the amniotic cavity, the extraembryonic coelom and eggshell, it is difficult for the embryo to encounter external antigens. Therefore, TNF-α does not function during the early development of the chicken embryo, displaying low mRNA expression at E8, E11 and E17, and this finding is associated with the methylation levels of the *TNF-α* promoter, which first increase and then decrease, and the more limited increase in genomic DNA methylation observed in the present study. During late embryonic development, the embryonic volume and air chamber continuously grow, the eggshell becomes thinner, and the breathing pattern of the chick embryo changes to pulmonary respiration. It is therefore easier for the embryo to become infected with external antigens. To face these challenge, the chicken embryo requires higher expression levels of TNF-α and decreased promoter methylation, which can be observed at E17 in the current study.

Insulin-like growth factor 2 (IGF2) serves a critical function in cell proliferation and division and the basal metabolic regulation of the embryo [40]. It is more strongly associated with embryogenesis and remains at a high level in organs throughout the growth of the embryo [41–43]. Previous results showed that the mRNA expression of *IGF2* and its receptor can even be detected in mouse two-cell embryos [44]. In this study, *IGF2* also showed increasing expression in the muscle and liver of the chicken embryo. It is well known that promoter DNA hypomethylation leads to high gene expression; however, little is known regarding the role of the methylation status of the gene body in gene expression. To examine the underlying cause of the incongruity among the decreasing methylation of the *IGF2* promoter, the hypomethylation of the *TNF-α* promoter in late embryonic development and the gradually increasing levels of genomic DNA methylation found in this study, we analyzed the methylation status of the *IGF2* and *TNF-α* gene bodies. Interestingly, the methylation levels of the *IGF2* gene body increased significantly with increasing embryonic age, and *TNF-α* exhibited a higher level of gene body methylation at E17, compared with the other embryonic ages.

DNA methylation is unevenly distributed throughout the genome: the highest levels are detected in repetitive sequences, heterochromatin and gene bodies, whereas the 5' and 3' flanking regions of genes maintain lower methylation levels [32,45–47]. The promoter methylation status is widely acknowledged to control gene expression. Once methylation has occurred in a gene promoters, methylated CpG is marked by H3K9me3 and then bound by methyl-CpG-binding protein 2, resulting in transcription inhibition [48]; in contrast, DNA methylation of gene bodies might serve alternative functions and could improve translational efficiency [16,49]. Previous studies have indicated that the active X chromosome displays more-than-two-fold higher allele-specific methylation of the inactive X, and this methylation is concentrated on the gene bodies [50]. It has also been shown that DNA hypermethylation may contribute to the genomic stability and structural integrity of chromosomes [13].

The available evidence indicates that gene body hypermethylation is not necessarily associated with repression of transcription. The tertiary structure of the chromosome, which may hinder mRNA transcription, is presumably improved by DNA methylation of the gene body, resulting in enriched DNA methylation in the gene body regions, as was also found in the present study. Gene body-specific hypermethylation on the active X chromosome [50] and low genomic DNA methylation levels in many transcriptionally inactive genes suggest that additional epigenetic mechanisms contribute to the regulation of gene expression in a combinatorial and coordinated manner [13,51]. The results of the present study demonstrate that gene expression may be synchronously regulated by hypomethylation of the gene promoter and hypermethylaiton of the gene body. In this study, the genomic DNA methylation levels in the liver were markedly higher than those in the heart and muscle, particularly after E13. It is clear that higher methylation levels in the gene body contribute more to the increase in genomic DNA methylation because the liver is the center of biochemical metabolism and presents higher gene expression activity. However, DNA methylation of gene bodies has consequences beyond its recognized effects on gene expression, and further research on this topic is needed [52].

In addition, we analyzed the expression of *DNMT1*, *DNMT3a* and *DNMT3b* during embryonic development of chicken. DNMTs catalyze maintenance methylation (DNMT1) [53] and *de novo* methylation (DNMT3a and 3b) [54] throughout embryogenesis in birds. DNMT1, DNMT3a and DNMT3b are strongly expressed after implantation, when genome-wide remethylation of DNA occurs [55,56]. A previous study showed that genetic disruption of both DNMT1 and DNMT3b almost eliminate DNA methyltransferase activity and reduce the genomic DNA methylation level by more than 95% [57]. In this study, these three methyltransferases showed increases in the liver or/and muscle over the course of embryonic development of chicken, suggesting that maintenance methyltransferases cooperate with the *de novo* methylation methyltransferases to maintain the genomic DNA methylation levels.

The chicken (*Gallus gallus*), which bridges mammals and vertebrates in evolution, is usually used as a model species for studies of embryology [32,58]. Although mostly descriptive, the results presented herein provide the first data on variations in genomic DNA methylation and the DNA methylation status of specific genes during broiler embryogenesis. These findings may help improve the understanding of the methylation patterns during the embryonic development of birds and may lay a theoretical foundation for poultry epigenetics, particularly for studies focusing on the embryonic period.

Obviously, the present study has some limitations. First, we did not obtain the genomic DNA methylation status of chicken embryo tissues at E0 (before incubation) and E1. In mammals, after fertilization, the methylation levels of the zygote gradually decrease to the lowest levels at the early blastocyst period, and *de novo* methylation then occurs in somatic cell lineages, until these develop into mature somatic cells with a stable methylation status [59]. The chicken embryo undergoes the blastula stage before E2; thus, we may have failed to detect the baseline genomic DNA methylation levels. Second, we did not detect the mRNA and protein levels of genes, with the exception of *IGF2* and *TNF-α*, to verify the relationship between gene function and DNA methylation. Therefore, additional follow-up studies are needed.

In summary, the results obtained in the current study indicate that the genomic DNA methylation levels of chicken present an overall gradual increase during embryonic development, and that the process can be divided into three distinct stages. The genomic DNA methylation levels in tissues are related to the genes expression levels, and gene expression may be simultaneously regulated by hypomethylation of the promoter and hypermethylation of the gene body.

## Supporting Information

S1 Fig Result of DNA digestion by enzymes. (DOC)

S2 Fig Chromatograms of deoxycytidine and 5-methyl-2'-deoxycytidine standard samples. (DOC)

S3 Fig Chromatogram of DNA samples. (DOC)

S4 Fig Standard curves of deoxycytidine and 5-methyl-2'-deoxycytidine. (DOC)

S1 Table The average recovery of deoxycytidine and 5-methyl-2'-deoxycytidine. (DOC)

S2 Table Information of the antibodies used in the Western blotting analysis. (DOC)
