# Supplementary material for: DNA Methylation Variation Trends during the Embryonic Development of Chicken
Source: PLoS One. 2016 Jul 20;11(7):e0159230. doi: 10.1371/journal.pone.0159230 (PMC4954715; doi:10.1371/journal.pone.0159230)
Supplement: S1 Fig — (DOC) [file pone.0159230.s001.doc]

**S1 Fig.** **Result of DNA digestion by enzymes.**

**
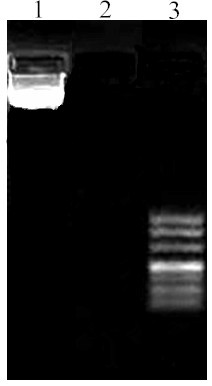
**

1. DNA extracted from tissues; 2. DNA digested by enzymes; 3. 500 bp DNA maker
