# Supplementary material for: DNA Methylation Variation Trends during the Embryonic Development of Chicken
Source: PLoS One. 2016 Jul 20;11(7):e0159230. doi: 10.1371/journal.pone.0159230 (PMC4954715; doi:10.1371/journal.pone.0159230)
Supplement: S2 Fig — (DOC) [file pone.0159230.s002.doc]

**S2 Fig. Chromatograms of deoxycytidine and 5-methyl-2'-deoxycytidine standard samples.**


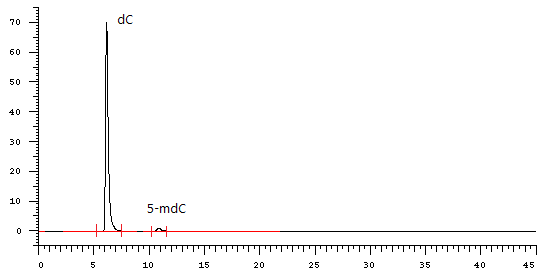


Retention time/min

Signal intensity/mv

dC: deoxycytidine; 5-mdC: 5-methyl-2'-deoxycytidine
