# Supplementary material for: DNA Methylation Variation Trends during the Embryonic Development of Chicken
Source: PLoS One. 2016 Jul 20;11(7):e0159230. doi: 10.1371/journal.pone.0159230 (PMC4954715; doi:10.1371/journal.pone.0159230)
Supplement: S3 Fig — (DOC) [file pone.0159230.s003.doc]

**S3 Fig. Chromatogram of DNA samples.**

Signal intensity/mv

Retention time/min


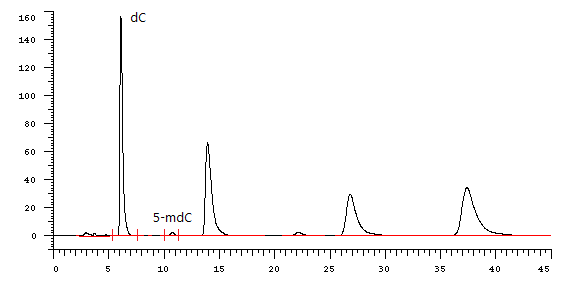


dC: deoxycytidine; 5-mdC: 5-methyl-2'-deoxycytidine
