# Supplementary material for: DNA Methylation Variation Trends during the Embryonic Development of Chicken
Source: PLoS One. 2016 Jul 20;11(7):e0159230. doi: 10.1371/journal.pone.0159230 (PMC4954715; doi:10.1371/journal.pone.0159230)
Supplement: S4 Fig — (DOC) [file pone.0159230.s004.doc]

**S4 Fig. Standard curves of deoxycytidine and 5-methyl-2'-deoxycytidine.**


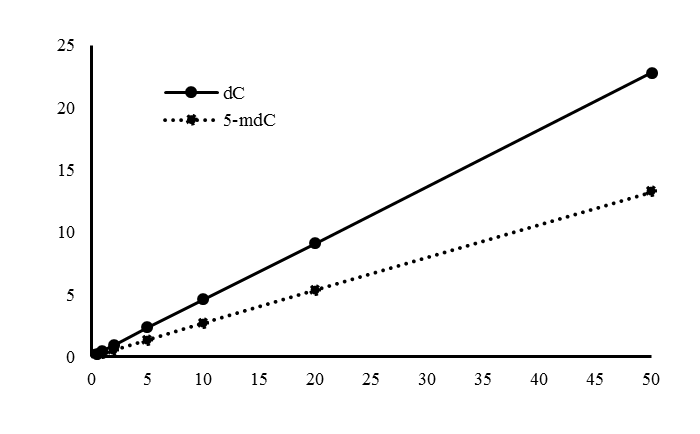


y=4.5517e+004x+2.825e+003

R² = 0.99998

y=2.6461e+004x+6.49e+002

R² = 0.99998

Concentration（mg/L）

Peak area/(×10^5^ mv·min)

dC: deoxycytidine; 5-mdC: 5-methyl-2'-deoxycytidine
