# Supplementary material for: DNA Methylation Variation Trends during the Embryonic Development of Chicken
Source: PLoS One. 2016 Jul 20;11(7):e0159230. doi: 10.1371/journal.pone.0159230 (PMC4954715; doi:10.1371/journal.pone.0159230)
Supplement: S1 Table — (DOC) [file pone.0159230.s005.doc]

**S1 Table. The average recovery of deoxycytidine and 5-methyl-2'-deoxycytidine (n=3, %).**

| Samples | Concentration/(mg/L) | | | | |
| --- | --- | --- | --- | --- | --- |
|  | 1 | 2 | 5 | 10 | 20 |
| dC | 94.6±4.1 | 95.7±5.2 | 98.1±7.9 | 97.3±9.8 | 101.2±3.4 |
| 5-mdC | 92.8±6.2 | 93.9±3.5 | 94.9±5.9 | 98.8±8.0 | 99.3±11.3 |

dC: deoxycytidine; 5-mdC: 5-methyl-2'-deoxycytidine
