# Supplementary material for: DNA Methylation Variation Trends during the Embryonic Development of Chicken
Source: PLoS One. 2016 Jul 20;11(7):e0159230. doi: 10.1371/journal.pone.0159230 (PMC4954715; doi:10.1371/journal.pone.0159230)
Supplement: S2 Table — (DOC) [file pone.0159230.s006.doc]

**S2 Table. Information of the antibodies used in the Western blotting analysis.**

| Antibodies | MW | Species | Source | Catalogue no. | Dilution ratio |
| --- | --- | --- | --- | --- | --- |
| IGF2 | 20 kDa | Rabbit | Abcam | Ab9574 | 1:1000 |
| TNF-α | 16 kDa | Rabbit | Abcam | Ab6671 | 1:1000 |
| DNMT3a | 85 kDa | Rabbit | Abcam | Ab16704 | 1:1500 |
| DNMT3b | 97 kDa | Mouse | Santa Cruz | sc-52922 | 1:1500 |
| β-actin | 43 kDa | Mouse | CWBIO | CW0096 | 1:3000 |
